# Fungal Cytochrome P450s and the P450 Complement (CYPome) of *Fusarium graminearum*

**DOI:** 10.3390/toxins10030112

**Published:** 2018-03-07

**Authors:** Jiyoung Shin, Jung-Eun Kim, Yin-Won Lee, Hokyoung Son

**Affiliations:** Department of Agricultural Biotechnology and Research Institute of Agriculture and Life Sciences, Seoul National University, Seoul 08826, Korea; sydsud@snu.ac.kr (J.S.); wjddms80@snu.ac.kr (J.-E.K.); lee2443@snu.ac.kr (Y.-W.L.)

**Keywords:** cytochrome P450, secondary metabolism, xenobiotics, *Fusarium graminearum*

## Abstract

Cytochrome P450s (CYPs), heme-containing monooxygenases, play important roles in a wide variety of metabolic processes important for development as well as biotic/trophic interactions in most living organisms. Functions of some CYP enzymes are similar across organisms, but some are organism-specific; they are involved in the biosynthesis of structural components, signaling networks, secondary metabolisms, and xenobiotic/drug detoxification. Fungi possess more diverse CYP families than plants, animals, or bacteria. Various fungal CYPs are involved in not only ergosterol synthesis and virulence but also in the production of a wide array of secondary metabolites, which exert toxic effects on humans and other animals. Although few studies have investigated the functions of fungal CYPs, a recent systematic functional analysis of CYP genes in the plant pathogen *Fusarium graminearum* identified several novel CYPs specifically involved in virulence, asexual and sexual development, and degradation of xenobiotics. This review provides fundamental information on fungal CYPs and a new platform for further metabolomic and biochemical studies of CYPs in toxigenic fungi.

## 1. Introduction

Cytochrome P450s (CYPs), which are heme-containing proteins, represent one of the largest protein families; they are present in all biological kingdoms [1,2]. CYPs commonly act as terminal monooxygenases in a range of biochemical reactions including hydroxylation, dealkylation, epoxidation, deamination, desulfuration, dehalogenation, sulfoxidation, and *N*-oxide reduction, by catalyzing the transfer of molecular oxygen to various cellular substrates [3,4,5]. This diversity of catalytic capabilities and the ability to manipulate them means that CYPs have been under the spotlight for biotechnological applications, such as biosynthesis of useful chemical compounds [6].

Organisms vary in the number of CYP genes they possess. The reference human genome contains 57 CYP genes and 58 pseudogenes, which are distributed into 18 families [7]. The function of human CYPs has been extensively studied since 1960, particularly with respect to xenobiotic and drug metabolism [8]. Insect genomes contain various numbers of CYPs, for example, 76–91 in *Drosophila* spp. (fruit flies), 87 in *Bombyx mori* (silkworm), and 46 in *Apis mellifera* (honey bee). In insects, CYPs are involved in the production of defence toxins and pheromones [9,10]. CYP-mediated detoxification of plant compounds, as well as insecticides, has also been reported [11]. Plant genomes tend to contain many more CYP genes than animal genomes, e.g., 334 in *Oryza sativa* (rice), 245 in *Arabidopsis thaliana* (thale cress), and 318 in *Zea mays* (corn), possibly because plants produce a wide variety of secondary metabolites [9]. Plant CYPs are mainly involved in biosynthesis of protective toxins and repellent molecules, as well as various signalling molecules [12].

The number of CYP genes in fungal species varies depending on their lifestyle. Whereas yeast-like fungi possess relatively few CYPs (three in *Saccharomyces cerevisiae*, six in *Cryptococcus neoformans*, and 10 in *Candida albicans*), filamentous fungi tend to possess more CYP genes. Plant pathogenic fungi tend to possess larger numbers of CYP genes; for example, *Magnaporthe oryzae* and *Cryphonectria parasitica* harbor 107 and 121 CYPs, respectively [13,14]. Fungal CYPs are involved in diverse biological processes, including production of primary and secondary metabolites and denitrification. However, compared to plants and animals, few fungal CYPs have been functionally characterized. Investigation of fungal CYPs will improve our understanding of fungal biology and metabolism, and may offer opportunities to exploit their catalytic functions.

In this review, we provide an overview of the fungal metabolic systems in which CYPs play roles, such as primary and secondary metabolism, degradation of xenobiotics, and other fungal traits. We also present a phenome-based functional analysis of whole CYP genes in the plant pathogenic fungus *Fusarium graminearum,* which illuminates the functional diversity and potential applications of fungal CYPs.

## 2. Fungal CYPs

Fungi represent a large kingdom of lower eukaryotic organisms, which are ubiquitous in ecological niches such as soil, living plants and animals, and decaying organic materials [15]. To rapidly adapt to environmental stresses and new niches, fungi have evolved extraordinary cellular defense systems, including CYP-mediated mechanisms for detoxification of exogenous toxic compounds. In particular, filamentous fungi have an outstanding ability to degrade a variety of toxic substances (e.g., environmental pollutants, xenobiotics, and plant-derived toxins) [16,17,18,19], and some filamentous fungi are well known for production of characteristic toxins via CYPs. Recent genetic evidence suggests that CYP enzyme reactions are closely involved in fungal developmental processes and pathogenesis [20,21].

CYP nomenclature is mainly based on amino acid sequence identity; 40% identity or greater places CYPs in the same family and greater than 55% identity places CYPs in the same subfamily [7]. As mentioned above, CYPs are key enzymes in many fungal processes, and classifiable into multigene families, CYP51–CYP69, CYP501–CYP699, and CYP5001–CYP6999 [15,22,23] (Table 1). CYP51, CYP56, CYP61, and many other known fungal CYPs are involved in biosynthesis of primary and secondary metabolites, as well as detoxification/degradation of xenobiotics. Many studies have predicted the functions of CYPs of individual fungi using bioinformatics tools [24,25,26,27,28]. Fungal CYPs are grouped in 15 clades based on their phylogenetic relationships [14]. Clade 8 is composed of the most family members including CYP59, CYP60, and CYP65 (Table 1). Recently, 14,896 CYPs were identified from 157 fungal and oomycete species [29]. However, the precise biological functions of most fungal CYPs remain undefined. For example, the CYPome of the white rot fungus *Phanerochaete chrysosporium* comprises ~150 CYPs, mostly arranged in gene clusters. In this fungus, except for the structurally and functionally conserved fungal CYP families, CYP51, CYP61, and CYP53, the roles of the other CYPs are still largely unknown and await functional characterization [30,31].

## 3. CYPs Related to Secondary Metabolite Biosynthesis

**Figure 1:**
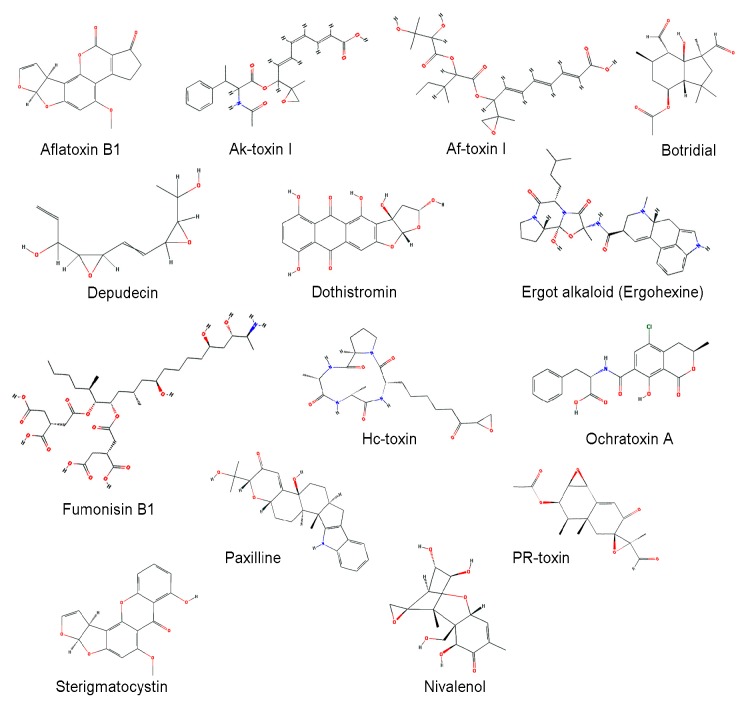
Chemical structures of mycotoxins. Chemical structures were obtained from PubChem (https://pubchem.ncbi.clm.cih.gov) [117].

Fungi produce a variety of secondary metabolites. In limited environmental niches, some fungi utilize secondary metabolites as weapons to compete against other organisms including bacteria, plants, animals, and even other fungi, and some toxins of pathogenic fungi function as important virulence factors in host-microbe interactions [91,92,93,94]. The functions of many fungal secondary metabolites remain obscure or unknown, but are predicted to play roles in interactions with other organisms. Secondary metabolites carry out a broad range of useful antibiotic, immunosuppressant, and mycotoxic activities [95,96,97]. Mycotoxins are toxic secondary metabolites produced by fungi, usually found in contaminated crops, and have severe effects on both humans and animals (Table 2). Structures of major mycotoxins are shown in Figure 1. Many fungal CYPs are known to be involved in mycotoxin biosynthesis [15,98].

### 3.1. Aflatoxins and Sterigmatocystin

Aflatoxins B1 (AFB_1_), B2 (AFB_2_), G1 (AFG_1_), and G2 (AFG_2_) and sterigmatocystin are polyketides derived from the same secondary metabolite biosynthetic pathway [118]. They are produced by several fungi, primarily by *Aspergillus* spp., which grow in soil, decaying vegetation, hay, and grain [119]. Although sterigmatocystin, the precursor of aflatoxin B1, has lower toxicity than other aflatoxins, both mycotoxins are potent carcinogens that cause liver cancer in mammals [120]. Among the 26 genes located in the aflatoxin biosynthetic gene cluster, *aflG*, *aflQ*, *aflU*, and *aflV* encode CYPs [76,99]. *aflG* is involved in the monooxygenase step that converts averantin to hydroxyaverantin, a precursor of aflatoxins [121]. *aflQ* encodes an oxidoreductase that is responsible for conversion of dihydro-*O*-methylsterigmatocystin to AFB_1_ and AFG_1_ and of *O*-methylsterigmatocystin to AFB_2_ and AFG_2_ [122,123]. *aflV* is involved in the reaction from averufin to 1′-hydroxyversicolorone, the first step in dihydrobisfuran formation during aflatoxin biosynthesis [74]. The pathway involvement of *aflU* remains unclear [99].

### 3.2. Fumonisins

Fumonisins are polyketide mycotoxins that cause severe animal diseases, including leukoencephalomalacia in horses, pulmonary edema in swine, and kidney and liver cancers in mice [124]. In addition, the consumption of fumonisin-contaminated maize induces high incidences of esophageal cancer in humans [125]. These mycotoxins are mainly produced by the maize pathogen *F. verticillioides* and several other *Fusarium* spp. [109]. At least 8 genes involved in biosynthesis of this toxin have been identified, one of which, *FUM6*, encodes a CYP [88]. In the fumonisin backbone, C-14 and C-15 are probably the direct substrate for Fum6-catalyzed hydroxylation [109,126].

### 3.3. Host-Selective Toxins

Host-specific toxins (also known as host-selective toxins; HSTs), which are produced by a number of plant pathogenic fungi, are a class of low-molecular-mass secondary metabolites [127]. They are called HSTs because these toxins are critical determinants of pathogenicity in specific plant–disease interactions [127]. The genera *Alternaria* and *Cochliobolus* are well known to produce HSTs [128]. *A. alternata*, a pathogenic fungus on a number of plants, produces several structurally diverse HSTs; AF-, AK-, ACT-, and AAL-toxins are produced by the strawberry, Japanese pear, tangerine, and tomato pathotypes of *A. alternata*, respectively [129]. In the Japanese pear pathotype, *AKT7* encodes a CYP that suppresses AK-toxin production [100]. There are 13 *ALT* genes involved in the biosynthesis of AAL-toxin, and *ALT2*, a homolog of *FUM6*, also encodes a CYP [129].

The cyclic tetrapeptide HC-toxin is an inhibitor of histone deacetylases in several organisms, including plants and animals [130]. It is also a well-known host-selective toxin produced by *C. carbonum* that selectively affects maize lines of genotype *hm1*/*hm1* [127]. One of the genes for HC-toxin biosynthesis encodes a CYP, which is involved in generation of the epoxide group [111].

### 3.4. Dothistromin

Dothistromin (DOTH), a polyketide-derived mycotoxin produced by *D. septosporum*, has broad-spectrum toxicity to plants, animals, and microbial cells via generation of oxygen radicals [131]. *D. septosporum* is an important forest pathogen that causes red band needle blight disease of pine trees [132], and DOTH is a virulence factor that affects the severity of disease, even though it is not required to cause disease [133]. The structure of DOTH is similar to that of versicolorin B, a precursor of aflatoxin, and the two DOTH biosynthetic CYP genes, *CypX* and *AvnA*, are orthologs of *aflV* and *aflG*, respectively. AvnA catalyzes the conversion of averantin to hydroxyaverantin in the early part of the DOTH biosynthetic pathway [106].

### 3.5. Botridial

*B. cinerea* is the causal agent of gray mold disease in more than 200 crop species worldwide [134]. *B. cinerea,* which has a necrotrophic lifestyle, secretes diverse cell wall–degrading enzymes and toxins to kill host cells [135]. Botridial is the best-studied toxin produced by this fungus, and CYP BcBot1 is responsible for hydroxylation of the C-15 carbon of 10-hydroxyprobotryane during botridial biosynthesis [103].

### 3.6. Ochratoxin A

Ochratoxin A (OTA) is a naturally occurring mycotoxin produced by a few of *Aspergillus* and *Penicillium* spp., e.g., *Aspergillus ochraceus*, *A. carbonarius*, *A. westerdijkiae*, *A. steynii* and *Penicillium verrucosum*, *P. nordicum*, and *P. thymicola*, on improperly stored food products [112,136]. OTA is the most toxic and frequently found among several forms of ochratoxins. The International Agency for Research on Cancer (IARC) has classified OTA as a possible human carcinogen, based on demonstrated carcinogenicity in animal studies [137]. Unlike other well-characterized mycotoxins, ochratoxin biosynthetic pathway has not yet been unraveled in detail. In addition to polyketide synthases (PKSs) and non-ribosomal peptide synthases (NRPSs), several key genes required for biosynthesis of OTA including putative CYPs were identified in a few *Aspergillus* species. Moreover, conserved OTA biosynthetic genes have been recently identified using a comparative genome analysis in several *Aspergillus* and *Penicillium* species [138].

## 4. Xenobiotic-Metabolizing CYPs

The environment contains a great number of substances and their mixtures, and more than 135 million organic and inorganic chemicals have been registered in the CAS Registry^SM^ collection to date [139]. Chemical compounds found within an organism or ecosystem that are not produced by that organism or ecosystem are called xenobiotics. Many xenobiotics are usually synthesized for industrial and agricultural purposes, i.e., aromatics, pesticides, and hydrocarbons; some of them are harmful to living organisms. Organisms have evolved efficient systems to prevent absorption of xenobiotics, to eliminate them, and to repair or adapt to damage caused by xenobiotics. Among xenobiotic-metabolizing enzymes, CYPs are the most abundant and versatile [140,141].

Studies of mammalian xenobiotic-metabolizing CYPs have led to the discovery and characterization of the xenobiotic metabolism pathways [8,140,141]. In humans, drug-metabolizing CYPs mostly presenting in the liver are responsible for oxidative metabolism of xenobiotics [140]. Many insect CYPs also play roles in detoxification of xenobiotics. Insect CYPs are inducible in response to botanical insecticides and/or plant secondary metabolites [11,142,143]. Also, some plant CYPs involved in the detoxification of xenobiotics such as herbicides [9]. In fungi, the wood-rotting basidiomycetes, particularly the white rot fungus *P. chrysosporium* and the brown rot fungus *Postia placenta*, are the most well-known fungi involved in the biodegradation of various xenobiotic compounds [144,145]. In particular, in *P. chrysosporium* 33 CYP families are involved in the hydroxylation of polycyclic aromatic hydrocarbons, and the genes belonging to the CYP63 family are predicted to be involved in degradation of various xenobiotics [144].

One of the best-characterized fungal CYPs is a pisatin demethylase (PDA, CYP57A1), which was first identified in *N. haematococca* [146]. PDA is the enzyme responsible for detoxifying pisatin, one of the isoflavonoid phytoalexins produced by *Pisum sativum* L. (garden pea). *N*. *haematococca* isolates with pisatin demethylating activity are tolerant to pisatin and highly virulent on pea, suggesting that PDA is a host-specific virulence factor in this fungus. *F. oxysporum* f. sp. *pisi*, another pea pathogen, possesses orthologs of PDA that play a major role in pisatin detoxification [147].

## 5. CYPs Required for Fungal Development and Virulence

One particularly well-studied fungal CYPs is CYP51, which mediates primary metabolism of ergosterol biosynthesis [148]. Ergosterol is a fungal-specific membrane sterol required for regulation of membrane fluidity and permeability [148,149]. It is essential for fungal growth and therefore is a primary target of antifungal compounds. Therefore, CYP51 has been exploited as a disease control target of fungal pathogens [139,150]. Sterol 14α-demethylase, a member of the CYP51 family, is the main target for therapeutic azole antifungal drugs and agricultural azole fungicides. Although the precise biochemical mechanism remains unclear, CYP51 orthologs are responsible for sensitivity to different azole fungicides, as these compounds specifically bind and inhibit sterol 14α-demethylase paralogs. Most fungi, including *A*. *nidulans*, *A*. *fumigatus*, *C*. *albicans*, *C. neoformans*, as well as *F*. *graminearum,* possess one or multiple CYP51 genes [139,151,152].

Oxylipins are oxygenated metabolites of linoleic or oleic acids that have hormone-like functions in sexual and asexual reproduction in many fungi [153]. The precocious sexual inducer (psi) factor-producing oxygenases (Ppos), natural fusion proteins of a heme peroxygenase at *N*-terminus and a P450 domain at C-terminus, are required for the production of oxylipins in *Aspergillus* spp., and CYP52-mediated oxygenation of hydrocarbons or fatty acids is important for lipid metabolism and pathogenesis in the human pathogen *C*. *albicans*. However, their exact biochemical mechanisms remain unclear.

Along with CYP51, which encodes sterol 14α-demethylase, CYP56 of *S. cerevisiae* and *C*. *albicans* are necessary for production of *N*, *N*′-bisformyl dityrosine, a component of the outer spore wall [65]. However, a homolog of CYP56 in *C*. *albicans* was found not to be essential for cell viability under culture conditions [66].

Fungal denitrification is the major pathway of nitrogen cycle in nature. CYP55, which encodes nitric oxide reductase (P450nor), is considered essential for most fungal denitrifying systems [154]. The multifunctional detoxifying enzyme CYP55 catalyzes the co-denitrification reaction and additionally exhibits NADH-peroxidase activity. Homologs of the CYP55 gene (P450nor) are distributed in many fungal genomes.

Significant regulation of CYPs has been observed during host-pathogen interactions via gene/transcriptome profiling analyses of several fungi, e.g., *F*. *graminearum*, *Curvularia lunata*, and *Heterobasidion annosum*, although the mechanisms are not yet known [155,156,157]. Recently, a pathogenicity-related CYP gene has been identified in *Verticillium dahliae* via transfer DNA (T-DNA) random insertional mutagenesis [158].

## 6. CYPs of *F. graminearum*

**Figure 2:**
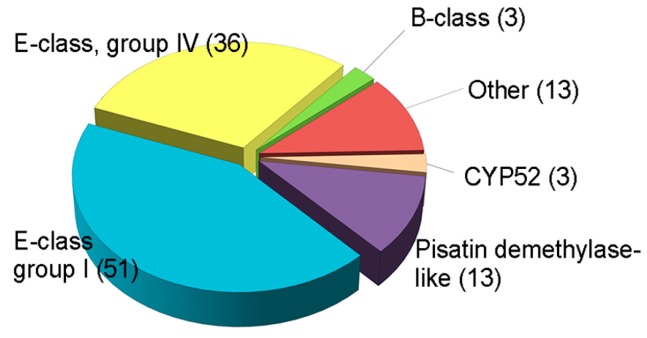
Classification of putative CYPs in *F*. *graminearum*. Total CYPs were categorized into six classes based on InterPro terms. These data were reproduced from [20]. Copyright 2017, John Wiley & Sons.

Several fungal CYPs have been known to play critical roles in primary and secondary metabolism and degradation of xenobiotics [159]. Besides, some CYPs in pathogenic fungi has emerged as important enzymes in virulence [155]. However, the involvement of CYPs in fungal development and other functions, such as virulence or xenobiotic detoxification, has rarely been elucidated, and there has been no systematic approach to the investigation of CYPs in filamentous fungi, including plant pathogens. The plant pathogenic fungus *F. graminearum* is an economically important pathogen that causes *Fusarium* head blight in major cereal crops such as wheat, barley, and rice, and *Fusarium* ear and stalk rot in maize worldwide [160]. In addition to yield losses, the fungus contaminates grains with mycotoxins such as trichothecenes (nivalenol (NIV) and deoxynivalenol (DON)) and zearalenone (ZEA), which pose serious threats to human and animal health [95]. *F. graminearum* is a good model organism, as its genome has been sequenced and targeted genetic modification is relatively easy [161]. The genome of *F. graminearum* is predicted to contain 119 putative CYP genes, which constitute 0.9% of the total predicted genes (Figure 2) [13].

**Figure 3:**
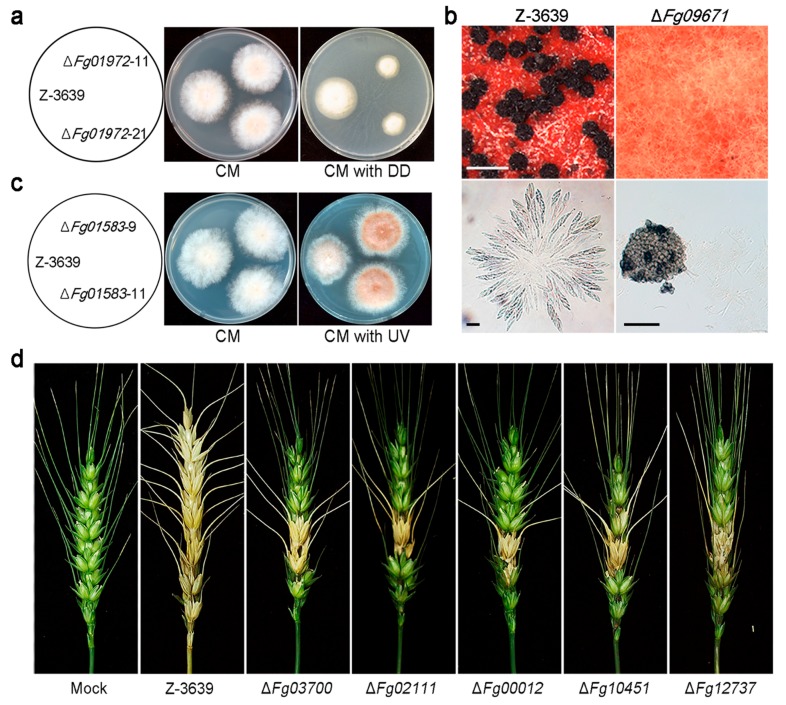
Phenotypic analyses of CYP deletion mutants of *F*. *graminearum*. (**a**) Altered xenobiotic stress response of wild type (Z-3639) and one CYP deletion mutant. DD, 1-dodecanol; (**b**) Development of perithecia (upper panel) and formation of asci rosettes (lower panel); A CYP gene deletion strain (right panel) showed defects in perithecia and ascospore formation whereas the parent strain (left panel) displayed normal perithecia and ascospores. Scale bar = 500 μm (upper), 20 μm (lower left), and 200 μm (lower right); (**c**) Altered ultra-violet (UV) stress response of wild type and CYP mutant strain; (**d**) Virulence of wild-type and CYP deletion strains on wheat heads. Five mutants showed reduced virulence compared to the wild-type strain (Z-3639). These data have been reproduced from [21] with slight modifications, Copyright 2013, John Wiley & Sons.

To date, only nine *F. graminearum* CYPs have been functionally characterized. Three CYP genes are paralogs of *CYP51,* which encodes an ergosterol biosynthetic enzyme [21,139]. Four CYPs (*TRI1*, *TRI4*, *TRI13*, and *TRI11*) are involved in trichothecene biosynthesis [71,162], and *Fg08079* and *CLM2* are required for the biosynthesis of butenolide [163] and culmorin [164], respectively. To systematically characterize the CYPome of *F. graminearum*, CYP gene knockout mutants were generated, and a comprehensive phenome of 102 CYP mutant library of *F. graminearum* was tested in 38 traits (Figure 3) [20]. Notably, specific CYP genes have been identified that are required for virulence (five CYPs), conidiation (one CYP), and sexual development (two CYPs) in this fungus.

### 6.1. Trichothecenes

**Figure 4:**
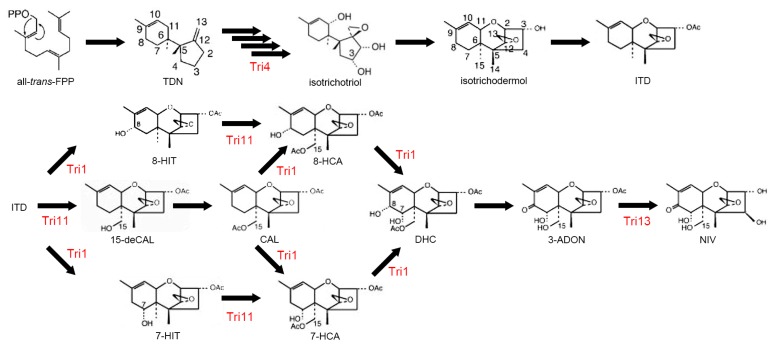
*Fusarium* trichothecene biosynthetic pathway. CYP enzymes, Tri1, Tri4, Tri11, Tri13, are involved in trichothecene biosynthesis. This scheme has been derived from [71] with slight modifications, Copyright 2007, Japan Society for Bioscience, Biotechnology, and Agrochemistry.

Trichothecenes are a large family of sesquiterpenoid secondary metabolites mainly produced by *Fusarium* spp. [165]. They are potent inhibitors of protein translation in eukaryotes via ribosomal binding [166]. DON and NIV are not only phytotoxins that contribute to the virulence *of F. graminearum*, but also mycotoxins that cause moldy-grain toxicosis in animals. In experimental animals, acute high-dose exposure induces a radiomimetic effect, with symptoms such as diarrhea, vomiting, leukocytosis, and gastrointestinal hemorrhage. At extremely high doses, these effects can cause circulatory shock, reduced cardiac output, and ultimately result in death [167]. NIV is significantly more toxic than DON [168]. Four CYPs, Tri1, Tri4, Tri11, and Tri13, are involved in biosynthesis of trichothecenes (Figure 4). Among the four CYPs in the trichothecene biosynthetic gene cluster, Tri13, 3-acetyltrichothecene C-4 hydroxylase, is responsible for hydroxylation of trichothecenes at C-4 [169]; the gene is therefore a determinant of the DON- and NIV-producing chemotypes in *F. graminearum* [170]. *TRI4* encodes a multifunctional oxygenase that is responsible for the conversion of trichodiene to isotrichotriol. *TRI4* mutants do not produce trichothecenes and highly accumulate trichodiene [70,171]. *TRI1* and *TRI11* encode 3-acetyltrichothecen C-8 hydroxylase and isotrichodermin C-15 hydroxylase, respectively [71].

### 6.2. Xenobiotic-Metabolizing CYPs in F. graminearum

CYPs have been extensively studied in many organisms because of their abilities for xenobiotic decomposition. Likewise, some putative xenobiotic-metabolizing CYPs in *F*. *graminearum* have been characterized based on genetic evidence [20]. *Fg01972* (CYP505A7) mutant strains showed reduced growth on 1-dodecanol-amended medium compared to the wild type (Figure 3a), and transcription of *Fg01972* was highly up-regulated in response to exogenous treatment with 1-dodecanol. The Fg01972 enzyme may play an important role in detoxification and/or mineralization of this compound. Moreover, most CYP genes (93 of 102) were markedly induced in at least one xenobiotic condition [20], demonstrating that many are closely related to xenobiotic metabolism, with redundant functions. Further studies are required to identify the substrates of these CYP enzymes.

In *F. oxysporum*, CYP505 members are fatty acid hydroxylases that perform subterminal omega hydroxylation of fatty acids [86]. Both the CYP505 (*Fg07596* and *Fg01972*) and CYP540 (*Fg02138* and *Fg02929*) families in *F*. *graminearum* are highly induced by aliphatics such as n-dodecane and 1-dodecanol [20]. In addition, Fg10451 belongs to the CYP53A subfamily hydroxylates benzoic acid and other monosubstituted benzoate derivatives, forming para-hydroxylated products in the ascomycete fungi *A. niger*, *A. nidulans,* and *C. lunatus,* as well as in the basidiomycete fungus *P. chrysosporium* [51,52,55,56]. *Fg02458* and *Fg00012* are members of the CYP63 family, which is predicted to take part in the degradation of fatty acids, and members of the subfamily CYP537A2 (*Fg12737*) are known to be related to benzoate 4-monooxygenase cytochrome P450 [14,29].

### 6.3. CYPs Required for Fungal Development and Virulence in F. graminearum

Phenotype-based screens of mutant libraries have provided a powerful approach for functional genomic studies in *F. graminearum* [161,172]. A comprehensive phenome set illuminated the molecular mechanisms underpinning sexual and asexual development, mycotoxin production, stress responses, and pathogenicity in this fungus [161]. Our previous study on the systematic functional characterization of CYP genes in *F. graminearum* revealed that many novel CYPs are closely involved in fungal developmental processes including virulence [20]. Using this phenotypic dataset, we could gain insight into the cryptic functions of CYPs in fungi.

In *A. nidulans*, the psi factor, hormone-like fatty acid-derived oxylipins, serves as a signal molecule that modulates sexual and asexual sporulation by affecting the timing and balance of asexual and sexual spore development [173]. *Fg09671* (CYP616A1) deletion mutants of *F. graminearum* showed defective sexual reproduction (Figure 3b) [20]. This indicates that *Fg09671*-mediated signalling might be related to regulation of the initial stages of sexual development, such as switching from mycelial growth to fruiting body development or differentiation of hyphae into fruiting body tissues.

The *Fg01583* (CYP642A1) deletion mutant produced orange pigment in mycelia and grew faster than the wild-type strain under ultra-violet (UV)-B conditions (Figure 3c). Most plants produce flavonoids as the major red, blue, and purple pigments, and these pigments play key roles in defense, as antimicrobial agents and UV protectants [174]. The exact biochemical function of *Fg01583* is not known yet, but this CYP642A1-type CYP enzyme seems to play a role in secondary metabolite biosynthesis, which is important for UV protection.

In infection assays with flowering wheat heads, deletion mutants of five CYPs, *Fg03700* (CYP620B1), *Fg02111* (CYP636A1), *Fg00012* (CYP630A1), *Fg10451* (CYP53A8), and *Fg12737* (CYP537A2), showed reduced virulence compared to the wild type (Figure 3d). Plants synthesize and accumulate secondary metabolites such as phytoalexins that are toxic to most fungi and are suspected of being involved in plant defence mechanisms [175]. However, fungal pathogens frequently possess the ability to detoxify host phytoalexins. For instance, PDA, a CYP enzyme produced by *N. haematococca*, detoxifies the pisatin produced by its host [16]. We suspect that these CYPs, which displayed reduced virulence when deleted, are involved in degradation of plant-derived metabolites or plant tissues for successful infection. Recently, comparative *in planta* transcriptome analyses revealed that more than 40 CYPs may be involved in host–pathogen interactions of *F*. *graminearum* [176].

## 7. Conclusions

Fungal CYPs play essential roles for survival, and several azole fungicides that are mainly targeted at the CYPs have been commercially used for control of animal and plant pathogenic fungi [177]. Decades of studies on fungal genetics and biochemistry have established the involvement of CYPs in many bioconversion processes related to degradation of foreign compounds and biosynthesis of secondary metabolites in fungi. However, scant information is available on CYPs and their involvement in fungal developmental processes, including virulence, due to the lack of CYP mutants defective in those phenotypes and available up-to-date molecular techniques. Using data from a systematic functional genomic study of *F. graminearum*, we identified some clues to the novel functions of CYPs in toxigenic fungi. Further studies should focus on the identification of the substrate specificity of CYPs using metabolomic and/or biochemical approaches. Moreover, the complex regulatory genetic networks governing CYP enzyme reactions will be uncovered by applying advanced molecular genetics techniques and multi-omics approaches. These multidisciplinary studies on CYPs will provide fundamental information on fungal-specific CYPs compared to those of other organisms and new insights into fungal biology and virulence.

## Figures and Tables

**Table 1:** Cytochrome P450 monooxygenases in fungi.

Clade ^1^	Family	Class ^2^	Organism	Function	Reference
1	CYP51	E, group I, IV	*S. cerevisiae*, *C. albicans*, *C. kefyr*, *C. glabrata*, *C. guilliermondii*, *C. parapsilosis*, *C. tropicalis*, *C. krusei*, *Ustilago maydis*, *Schizosaccharomyces pombe*, *Kluyveromyces marxianus*, *Penicillium italicum*, *Fusarium graminearum*	Demethylation of eburicol/lanosterol at 14α position	[21,23,32,33,34,35,36,37,38,39,40,41,42]
2	CYP52	E, group II	*C. maltose*, *C. tropicalis*, *C. apicola*	*n*-alkane and fatty acid assimilation	[3,42,43,44,45,46,47,48,49,50]
2	CYP53	E, group I	*Aspergillus niger*, *Beauveria bassiana*, *Cochliobolus lunatus, P. chrysosporium*, *Rhombophryne minuta*	Degradation or detoxification benzoate and its derivatives	[51,52,53,54,55,56]
2	CYP54	E, group I	*Neurospora crassa*	Cycloheximide inducible, but function is unknown	[57]
3	CYP55	E, group I	*F. oxysporum, Cylindrocarpon tonkinensis, A. oryzae*, *Trichosporon cutaneum*	Denitrification process	[58,59,60,61,62,63]
4	CYP56	E, group IV	*S. cerevisiae*, *C. albicans*	Formation of dityrosine	[64,65,66]
6	CYP57	E, group I	*Nectria haematococca*	Pisatin detoxification	[67,68,69]
6	CYP58	E, group I	*F. sporotrichioides*, *F. graminearum*	Trichothecene biosynthesis (*TRI4*)	[70,71]
7	CYP58	B	*A. flavus*, *A. parasiticus*	Aflatoxin biosynthesis	[72,73,74]
8	CYP59	E, group I	*A. nidulans*	Sterigmatocystin biosynthesis (*stcS*/*verA*)	[75,76]
8	CYP60	E, group I	*A. parasiticus*, *A. nidulans*	*o*-methylsterigmatocystin to aflatoxin (*ord1*), sterigmatocystin biosynthesis (*stcF* and *stcL*)	[76,77]
8	CYP61	E, group I	*S. cerevisiae*, *C. glabrata*	Sterol D22-desaturase in ergosterol biosynthesis (*erg5*)	[22,78]
8	CYP62	E, group I	*A. nidulans*	Sterigmatocystin biosynthesis (*stcB*)	[76]
8	CYP63	E, group I	*P. chrysosporium*	Unknown function	[79]
8	CYP64	E, group I	*A. flavus*	Conversion of *o*-methylsterigmatocystin to aflatoxin (*ord1*)	[80]
8	CYP65	E, group I	*F. sporotrichioides*	Trichothecene biosynthesis (*TRI11*)	[71]
9	CYP66	E, group IV	*Agaricus bisporus*	Developmental regulation of mushroom	[81]
10	CYP68, CYP69, CYP503	E, group I	*F. fujikuroi*	Gibberellin biosynthesis	[82,83]
10	CYP504	E, group I	*A. nidulans*	Catalyzing phenylacetate 2-hydroxylation	[84,85,86]
14	CYP505	E, group IV	*F. oxysporum*	ω-1 to ω-3 carbon hydroxylation of fatty acids	[86,87]
15	CYP505	E, group IV	*F. verticillioides*	Fumonisin biosynthesis	[88,89]
15	CYP526	E, group IV	*F. sporotrichioides*	Trichothecene biosynthesis	[71]

^1^ The fungal CYP families fall into 15 clades based on their phylogenetic relationships [14]; ^2^ This classification of CYPs is based on the number of components in the system [90].

**Table 2:** Mycotoxins produced by Cytochrome P450 (CYP)-mediated reactions.

Mycotoxin	Organism	Characteristics	Reference
Aflatoxin	*A. flavus*, *A. parasiticus*, etc.	Carcinogenic compounds posing a potential risk to livestock and human health	[99]
Ak-toxin	*Alternaria alternata*	Host-selective toxin, virulence factor to infect Japanese pear	[100]
Af-toxin	*A. alternata*	Host-selective toxin, virulence factor to infect strawberry	[101]
Botridial	*Botrytis cinerea*	Induction of chlorosis and cell collapse in plant	[102,103,104]
Depudecin	*A. brassicicola*	An inhibitor of histone deacetylase (HDAC)	[105]
Dothistromin	*Dothistroma* *septosporum*	A broad-spectrum toxin that generates oxygen radicals by reductive oxygen activation	[106]
Ergot alkaloid	*Claviceps*, *Penicillium*, and *Aspergillus* spp.	A complex family of indole derivatives with diverse structures and biological activities	[107,108]
Fumonisin	*F. verticillioides*	Induction of several animal diseases, including leukoencephalomalacia, pulmonary edema, and cancer	[109]
Hc-toxin	*Cochliobolus carbonum*	An inhibitor of histone deacetylases (HDACs) in many organisms, including plants, insects, and mammals	[110,111]
Ochratoxin	*Aspergillus,* and *Penicillium* spp.	Possible carcinogenic	[112]
Paxilline	*P. paxilli*	A potassium channel blocker	[113,114]
PR-toxin	*P. roqueforti*	Liver toxicity and abortions in cows	[115]
Sterigmatocystin	*A. nidulans*, *A. versicolor*	A toxic metabolite structurally closely related to the aflatoxins	[75,76]
Trichothecene	*F. sporotrichioides*, *F. graminearum*	Inhibition of protein synthesis and highly cytotoxic to many eukaryotes	[71,95,116]
